# Xer Site Specific Recombination: Double and Single Recombinase Systems

**DOI:** 10.3389/fmicb.2017.00453

**Published:** 2017-03-20

**Authors:** Fabio Castillo, Amal Benmohamed, George Szatmari

**Affiliations:** Département de Microbiologie, Infectiologie et Immunologie, Université de Montréal, MontréalQC, Canada

**Keywords:** site-specific recombination, tyrosine recombinases, single recombinases, XerS, XerH, XerA, IMEX

## Abstract

The separation and segregation of newly replicated bacterial chromosomes can be constrained by the formation of circular chromosome dimers caused by crossing over during homologous recombination events. In *Escherichia coli* and most bacteria, dimers are resolved to monomers by site-specific recombination, a process performed by two Chromosomally Encoded tyrosine Recombinases (XerC and XerD). XerCD recombinases act at a 28 bp recombination site *dif*, which is located at the replication terminus region of the chromosome. The septal protein FtsK controls the initiation of the dimer resolution reaction, so that recombination occurs at the right time (immediately prior to cell division) and at the right place (cell division septum). XerCD and FtsK have been detected in nearly all sequenced eubacterial genomes including Proteobacteria, Archaea, and Firmicutes. However, in S*treptococci* and *Lactococci*, an alternative system has been found, composed of a single recombinase (XerS) genetically linked to an atypical 31 bp recombination site (*difSL*). A similar recombination system has also been found in 𝜀-proteobacteria such as *Campylobacter* and *Helicobacter*, where a single recombinase (XerH) acts at a resolution site called *difH*. Most Archaea contain a recombinase called XerA that acts on a highly conserved 28 bp sequence *dif*, which appears to act independently of FtsK. Additionally, several mobile elements have been found to exploit the *dif*/Xer system to integrate their genomes into the host chromosome in *Vibrio cholerae, Neisseria gonorrhoeae*, and *Enterobacter cloacae*. This review highlights the versatility of *dif*/Xer recombinase systems in prokaryotes and summarizes our current understanding of homologs of *dif*/Xer machineries.

## Introduction

Bacteria and archaea have developed a variety of well-regulated and coordinated mechanisms of replication and segregation of their genomes that ensure the genetic material is transmitted faithfully to the daughter cells, despite the absence of temporal separation between DNA synthesis, chromosome separation and cell division (Reyes-Lamothe et al., 2012). However, the circular state of their chromosomes and plasmids constitutes a constant threat to genome stability and proper segregation because of dimer formation during recombinational exchanges between sister chromatids. These rearrangements can combine their genomes into larger molecules, compromising an equal distribution of the genetic material to the daughter cells (Barre et al., 2001; Yates et al., 2003; Massey et al., 2004; Grainge et al., 2007). This topological problem was fully addressed in 1981, when Austin et al. (1981) demonstrated that the stable inheritance of the prophage P1 was due to site-specific recombination (SSR), a specialized system that catalyzes DNA exchange between two defined DNA sequences, and which plays a major role in dimer resolution by converting multimeric forms to the monomeric forms. Later studies performed with the plasmid ColE1 connected SSR with plasmid monomerization and stability (Summers and Sherratt, 1984). Colloms et al. (1990), the site-specific recombinase (XerC) was identified as the first protein responsible for SSR on *cer*, followed by the identification of a second recombinase, XerD required for this reaction (Blakely et al., 1993).

## The Disadvantage of Having Circular DNA

In most bacteria and some archaea, replication begins at a single origin of replication *oriC* at which DnaA binds and stimulates the assembly of the replisome (Jha et al., 2016). Replication forks then proceed bi-directionally until the two replication forks meet in an antipodal terminus region flanked by *ter* sequences. These sequences in conjunction with the replication terminator protein (Tus) stop the replication forks to synchronize their arrival at the same time and place (Elshenawy et al., 2015; Gowrishankar, 2015; Pandey et al., 2015). However, chromosome replication is not a continuous process and is continuously halted by different types of DNA lesions such as UV irradiation, free radicals, genotoxic agents, DNA replication errors, transcription-replication conflicts, tightly bound protein-DNA complexes, or RNA secondary structures (Kuzminov, 1999; Tehranchi et al., 2010; Ayora et al., 2011; Merrikh et al., 2012; Gowrishankar, 2015). To maintain their genomic integrity, bacteria have developed several and sophisticated mechanisms to minimize the frequency of these DNA lesions before the occurrence of replication. The initial barrier against deleterious DNA modifications is carried out by specialized mechanisms, each one required for a given type of lesion, such as proofreading, direct reversal of DNA damage, base excision repair, nucleotide excision repair and mismatch repair (Lenhart et al., 2012; Kisker et al., 2013; Groothuizen and Sixma, 2015; van der Veen and Tang, 2015). Additional groups of mechanisms are responsible for avoiding transcription-replication encounters, equally lethal for bacteria, such as the coordination of temporal and spatial gene activation and co-orientation, modulators of RNA polymerases (RNAPs) and replicative accessory helicases (Pomerantz and O’Donnell, 2010; Merrikh et al., 2012; Bruning et al., 2015; Roghanian et al., 2015). Nonetheless, it is unavoidable that some of this DNA damage or conflicts will escape the initial barrier and interfere with replication fork migration, leading to the eventual inactivation of the replication machinery and formation of double-strand breaks (DSBs), interstrand cross-links and single-stranded gaps (SSG). These represent critical forms of DNA damage that must be removed for chromosome replication and transcription to proceed (Heller and Marians, 2006; Yeeles et al., 2013). Therefore, a second barrier of repair is called into play to cope with these “evasive” damages. This second barrier is preferentially carried out by the homologous recombination repair system (HR). Estimates indicate that HR repair is required in almost every cycle of replication (Cox et al., 2000; Vos and Didelot, 2009; Darmon et al., 2014). In fact, the HR system is now not only considered as a functional mechanism for generating genetic diversity but also as a decisive factor in DNA repair, the latter being the primary role of this system in the maintenance of the genome and the main source of dimer events (Rocha et al., 2005). Thus, HR plays a central role in removal and/or repair of DNA damage and rescue and/or re-assembling of replication forks that have been broken or stalled (Heller and Marians, 2006; Costes and Lambert, 2012). In the traditional HR system in *Escherichia coli*, its mode of action consists of a multistep process of breakage and rejoining of homologous sequences (one old and one newly synthesized DNA strand). It initially involves (1) recognition of the DNA lesion by the complexes RecBCD or RecFOR, depending on the type of DNA lesion; (2) formation of 3′-ssDNA overhangs processed by the exo and endonuclease activity of the Rec proteins, and subsequent coating by RecA; (3) strand invasion of the 3-terminal ssDNA into the homologous duplex DNA molecule and search of the complementary strand; (4) formation of a D-loop intermediate, transformation into a branched intermediate and Holliday junction (HJ) formation and (5) completion of the recombination process by resolution of the HJ, catalyzed by the systems RuvABC or RecG (Chen et al., 2008; Handa et al., 2009; Zhang et al., 2010). HJ resolution can result in two alternative products;

• ‘crossover’ or spliced products; where reassortment of the flanking genes of the cleavage site has occurred, obtaining one different genotype at one side compared to the former DNA duplex, and therefore, long range of genetic exchange.
• ‘non-crossover’ or patch products where the flanking regions were not exchanged, and instead, the resulting DNA duplex contain a ‘patch’ of hybrid DNA with a shorter range of genetic exchange (Cromie and Leach, 2000; Lilley and White, 2001).

**FIGURE 1:**
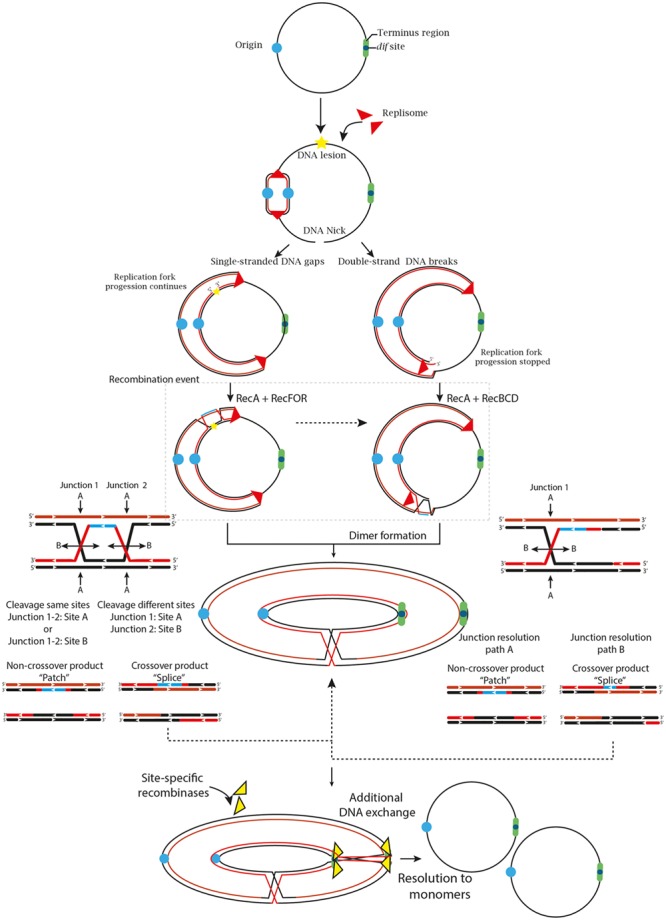
**The two primary and most generalized pathways to solve chromosomal dimers generated by RecA-dependent repair or stalled replication forks.** If the fork encounters a non-coding lesion (oxidative damage, pyrimidine dimer or an abasic site) depicted with a yellow star, it usually generates SSG (left path), although it can also lead to DSBs ends during repair by the RecFOR system. The pathways can also diverge on the location of the DNA lesion. If the lesion is located on the lagging strand template, the replisome will be able to bypass the lesion by blocking ‘Discontinuous DNA synthesis’ and then resume it downstream of the lesion, leaving a gap that would be repaired by the RecFOR system. On the other hand, a lesion on the leading strand template might transiently stop the replisome, cause its dissociation and then, bind further downstream to a new leading-strand primer, although these mechanisms are still under debate (Costes and Lambert, 2012; Yeeles et al., 2013). Alternatively, when the fork encounters a nick in the template strand (unrepaired SSG) or some cases of replication fork collapse, a DSB is generated (Right path). The DSBs are processed by the RecBCD complex that catalyzes the reattachment of the damaged DNA to the sister DNA duplex, forming a D-loop structure and eventual recognition by the replication-restart PriA protein that directs replisome assembly and resumption of the replication process in an origin-independent manner. However, odd numbers of crossover events generate dimer products (Yeeles and Dillingham, 2010; Azeroglu et al., 2016; Ðermić, 2015). Thus, if the resolution of the HJ occurs in the same sites, it will generate monomeric chromosome (Non-crossover products). In contrast, if the resolution takes place in different sites, it will generate chromosome dimers (Crossover products) (Carrasco et al., 2004; Ayora et al., 2011).

Because circular chromosomes do not have “ends,” they are vulnerable to concatenation during formation of an odd number of crossover events. Thus, swapping DNA flanking regions tangles the sister chromatids and forms larger ring chromosomes that compromise cellular division (**Figure 1**) (Steiner and Kuempel, 1998; Barre et al., 2001). To ensure proper chromosomal segregation, bacteria and archaea have overcome these major threats by two broad mechanisms. One is to minimize the formation of crossing-over events, and the other is to solve dimer formation by performing an additional DNA exchange, immediately prior to cell division, at a specific region called *dif* (Deletion-Induced Filamentation).

## Avoiding Dimer Formation

One way to avoid dimer formation, as simple as it sounds, is to decrease the likelihood of dimer formation. This, however, is an intricate process of coordination and selection of the right enzymes at the right moment. Therefore, if the resolution of HJ intermediates by endonucleolytic cleavage can only result in crossover or non-crossover products, the likelihood of obtaining one or another is 50%. However, minimizing crossover events during homologous recombination repair seems to be the rule rather than the exception in organisms with circular chromosomes (Martini et al., 2006). In *E. coli*, homologous recombination repair is processed by two predominant recombinational pathways; The RecBCD pathway associated with DSB repair, replication fork collapse, replication fork reversal and replication fork arrest, and the RecF pathway, which is mostly involved in the repair of SSG, and under certain conditions, can also repair DSBs (Labib and Hodgson, 2007; Johnston et al., 2015). Both mechanisms lead to the formation of HJs that are mostly resolved by the RuvABC complex in *E. coli* or RecU in Firmicutes and Mollicutes (Carrasco et al., 2004). Deletion of the genes of the RuvABC complex eliminates non-crossover formation bias, supporting the idea that bias formation mostly depends on the action of Ruv proteins more than Rec proteins (Cromie and Leach, 2000). This idea was initially discussed by Van Gool et al. (1999) who demonstrated that crossover and non-crossover products are not random and, conversely, they are influenced by the positioning and orientation of the resolvasome on the HJ intermediate, which in turn directs RuvC strand cleavage direction. Additionally, topological conditions such as DNA supercoiling, DNA catenation, adjacent HJ intermediates or the presence of double HJ intermediates can also influence assembly of the RuvABC complex on the HJ and indirectly affect resolution (Van Gool et al., 1999). Subsequently, Cromie and Leach (2000) showed that RuvABC positioning may depend on the nature of the substrate caused by the type of DNA lesion, thus DSBs are more likely to result in crossover products (Frequently processed by RecBCD) whereas SSG are more likely to result in non-crossover products (frequently processed by RecF). Although, some fractions of SSG can cause DSBs (Spies and Kowalczykowski, 2005). However, the specific causes of crossover or non-crossover formation are still under debate, and different reactions cannot be completely dismissed. A clear example of this is the fact that RecBCD as well as RecF are not restricted to DSBs and SSG respectively and, on the contrary, the both may have interchangeable functions (Pagès, 2016). That would explain why some replication fork arrests generate non-crossover products even if they are mostly processed by the RecBCD pathway (Michel et al., 2000), or why RecF contributes almost equally to dimeric chromosome formation in *E. coli* despite the fact that it is responsible for SSG resolution (Barre et al., 2001).

Interestingly, the fact that DNA lesions and transcription-replication conflicts are more abundant in the leading strand than in the lagging strand in *E. coli*, and that these lesions usually generate non-crossover products, reinforces the idea that organisms with circular chromosomes favored a system that minimizes dimer formation during HR repair completion (Cromie and Leach, 2000; Fijalkowska et al., 2012; Merrikh et al., 2012). These biased reactions have also been detected in other microorganisms such as *B. subtilis* where the resolvase RecU biases homologous recombination toward non-crossover products (Carrasco et al., 2004). Despite this non-crossover preference by HR system in *recA^+^* cells, dimer formation still occurs reaching 10–15% of the growing cells (Carnoy and Roten, 2009).

## Coping With Dimers

It is clear that dimer formation is regarded as a negative outcome that must be solved. Despite this, Mazin et al. (1996) proposed that under certain conditions of selective stress, plasmid dimerization could confer an advantage for the selection of adaptive mutations due to rapid accumulation and selection of plasmids carrying a specific mutation and subsequent segregation to the daughter cells. Berza et al. (2013) also reported that plasmid dimerization greatly increased synthesis of a foreign protein and that plasmid content is unaffected by dimer formation showing some advantages for transcriptional events. However, these benefits were only considered for plasmids. Regarding circular chromosomes, dimerization must be resolved by the action of site-specific recombinases (SSRs), which are enzymes that are responsible for breaking and rejoining specific sites without requiring DNA synthesis or high energy cofactors (Warren et al., 2013). The relevance of this system for proper chromosome segregation is supported by the high degree of conservation in Bacteria and Archaea. The Xer complex is considered one of the most conserved structural features in cells containing circular chromosomes, as well as RecA and FtsK enzymes (Carnoy and Roten, 2009; Kono et al., 2011; Diagne et al., 2014). The SSRs act on specific short DNA sequences, called recombination sites, where DNA exchange occurs in three different types of DNA rearrangements; deletion (divided into excision or resolution), insertion, or inversion. All these processes depend on the orientation and direction of the two recombination sites (Grindley et al., 2006) (**Figure 1**). All known site-specific recombinases are classified into two unrelated families, tyrosine–type or serine-type recombinases (Tyr or Ser) based on the amino acid residue that forms a covalent linkage between the protein and a phosphate at the DNA cleavage site (Hirano et al., 2011). Serine recombinases, often referred to as the resolvase/invertase family, act on a recombination site with just 2 bp separating the cleavage sites on top and bottom strands and the cleavages occur simultaneously to create a double strand break, while tyrosine recombinases, often referred to as the λ integrase family enzymes perform a two-step cleavage and rejoining process where the cleavage sites are separated by 6–11 bp. Each recombinase family possesses a distinct mechanism. Tyrosine recombinases are divided according to the recombination directionality; unidirectional or bidirectional recombinases. Whereas serine recombinases are divided according to their size; small or large recombinases (Grindley et al., 2006; Brown et al., 2011).

**FIGURE 2:**
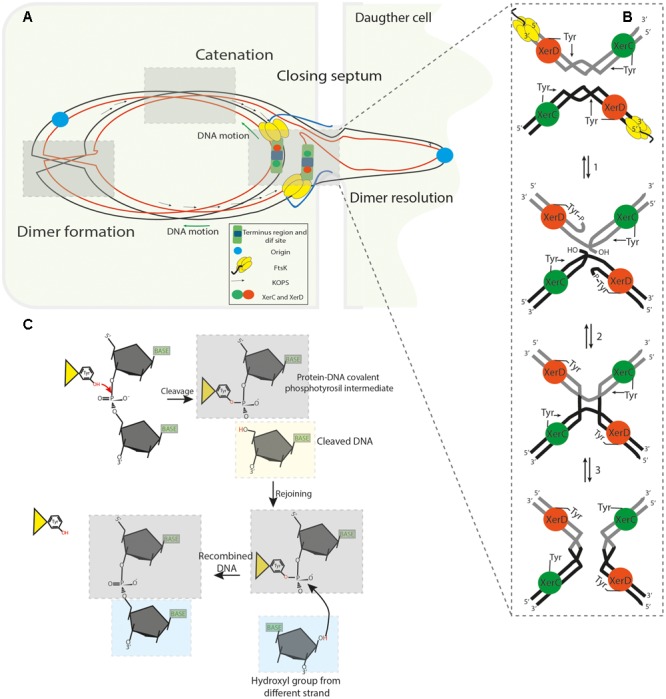
**(A)** Segregation of the sister chromatids during chromosome dimer resolution in *E. coli*. The illustration depicts the central part of a dividing cell in the final steps of chromosome segregation. The closing division septum, the motor domain αβ of FtsK_C_ (yellow hexameric ring), the unstructured linker domain FtsK_L_ (Blue ribbon), the KOPS sequences and the XerCD/*dif* synaptic complex are indicated. Concatenation prevents proper migration of the nascent chain of DNA; the origin regions move toward their respective cell poles, but the rest of the knotted DNA is stretched across and behind the septum. **(B)** FtsK_C_ loads onto the KOPS sequences in an oriented manner and translocates toward XerCD/*dif* complexes. FtsK translocation allows it to reach the XerCD/*dif* complexes and bring them into proximity; as a consequence, the γ-subdomain of the FtsK_C_ region activates XerD (Orange sphere) to perform the first strand cleavage. Then, XerC (Green sphere) mediates the second strand cleavage, allowing separation of the sister chromatids from each other. **(C)** Illustration of the SSR mechanism used by tyrosine recombinases: The OH group of the active residue tyrosine attacks the scissile phosphate forming a 3′-covalent phosphotyrosyl enzyme–DNA covalent intermediate and a free 5′-hydroxyl end. The covalent intermediate is attacked in turn by the other 5′- end to reverse the cleavage reaction and obtain a recombinant product.

**FIGURE 3:**
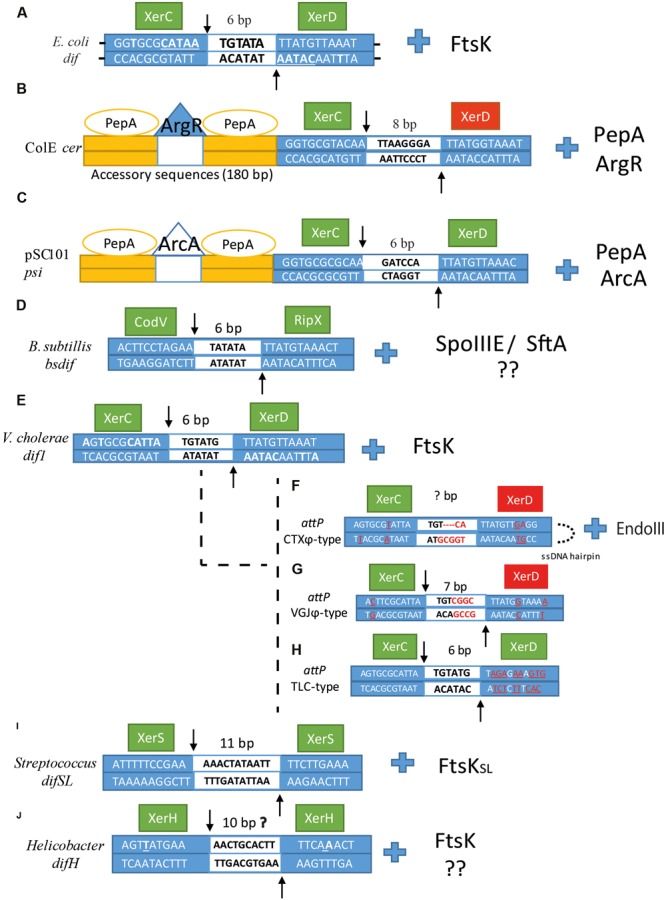
**Sequence alignment of (A)** *dif*, **(B)** *cer*, **(C)** *psi*, **(D)** *Bsdif*, **(E)** *dif1*, **(F)** *attP^CTX^*, **(G)** *attP^V GJ^*, **(H)** *attP^TLC^*, **(I)** *difSL* and **(J)** *difH.* Dyad bases in each arm are underlined in *dif*. XerC and XerD cleavage points are indicated by arrows, the central region is depicted in the middle of the sequence as a white box with the number of base pair corresponding to each *dif* site above. Left and right arms are depicted as blue boxes with their corresponding sequences for each *dif* site. The catalytic unit is depicted as a green box, whereas the inactivated unit is depicted as a red box. For **(E–H)**, bases that differ from *dif1* in *V. cholerae* are underlined and colored in red. Next to each *dif* alignment is the corresponding accessory protein that coordinates/activates dimer resolution.

The chromosome dimer resolution (CDR) process and heritable stability were originally elucidated in *E. coli* (Stirling et al., 1988a; Colloms et al., 1990; Blakely et al., 1993; Sherratt et al., 1995, 2004), where two paralogous site-specific tyrosine recombinases, XerC (298 aa) and XerD (298 aa) (Chromosomally Encoded Recombinases) were shown to act on a 28 bp DNA sequence (*dif* site), located in the *ter* region. The synaptic XerCD/*dif* complex consists of two XerC and two XerD subunits respectively bound to two *dif* sites (**Figure 2B**). Limited structural information of some tyrosine recombinases have revealed a conserved catalytic domain fold (Swalla et al., 2003), facilitating the analysis of experimental data and allowing the development of a general model for Xer recombinases (Meinke et al., 2016) consisting of; XerD (Subramanya et al., 1997), XerA (Hwa Jo et al., 2016), XerH (Bebel et al., 2016) and other related tyrosine recombinases like Cre (Gopaul and Van Duyne, 1999; Martin et al., 2002), HP1 integrase (Hickman et al., 1997), FLP (Chen et al., 2000) and λ integrase (Kwon et al., 1997; Biswas et al., 2005). The *E. coli dif* site is divided into two 11 bp half-sites that share partial dyad symmetry linked by a 6 bp central region that defines the positions of strand cleavage and exchange (Kuempel et al., 1991). The initial step of SSR during dimer resolution requires the formation of a synaptic complex consisting of a tetrameric protein/DNA complex (four protomers of tyrosine recombinases and two recombination site duplexes). Once the synaptic complex is formed, two opposing and activated protomers cleave the DNA strand of each recombination site duplex. This occurs when the hydroxyl group of the nucleophilic tyrosine attacks the scissile phosphate in the central region to form a 3′ phosphotyrosyl intermediate and a 5′-hydroxyl end. This intermediate conserves the energy from the phosphodiester bond cleavage to perform the first strand exchange. The recently formed 5′- hydroxyl attacks the 3′ phosphotyrosyl linkage on the partner site to reseal the strand breaks creating a HJ intermediate (Warren et al., 2013). HJ formation and isomerization activates the second pair of subunits bound to the other half of the recombination sites and inactivates the first pair of subunits. The second pair of subunits then cleaves, exchanges and rejoins the second pair of strands by the same mechanism just described; this second cleavage allows the resolution of HJ-intermediate and results in the recombinant DNA (**Figure 2B**) (Rajeev et al., 2009; Meinke et al., 2016). This process implies that the specific pairs of recombinases and/or active sites are continuously switched on and off to synchronize when and how recombination occurs, this coordination depends on allosteric interactions between the recombinases and external factors imposed on the synaptic complex (Hallet et al., 1999; Aussel et al., 2002; Vanhooff et al., 2009). In the XerCD/*dif* system, XerC normally initiates catalysis of one pair of DNA strands to form the HJ-intermediate without a subsequent resolution by XerD. Therefore, the HJs are rapidly converted back to the original DNA rearrangement. This XerC-first interaction is functionally active during the integration of certain bacteriophages that utilize Xer recombination to integrate their genomes into their host *dif* sites or in the resolution of plasmid multimers. In contrast, during chromosomal dimer resolution, pre-synapsed XerCD/*dif* complexes favor XerD activation by the FtsK protein to mediate the first strand exchange, generating a transient (XerD-HJ) intermediate, subsequent isomerization forms a XerC-HJ intermediate that is rapidly resolved to recombinant DNA by XerC, see below and **Figures 2A, 3A** (Zawadzki et al., 2013; Diagne et al., 2014).

## Establishing Rules for Dimer Resolution

One of the fundamental questions about SSR at *dif* concerns how the system is controlled to ensure a proper CDR into monomers in the right place and at the right time without promoting the reverse reaction, which would generate dimers from monomers. It is understood that Xer-mediated recombination mostly depends on an active HR system because it is the major process that provides concatenated chromosomes. However, catenation problems caused by replication may require Xer-recombination system as well (Recchia et al., 1999; Grainge et al., 2007; Midonet et al., 2014). This reaction occurs at two polarized and specific regions of ∼10 kb at either side of *dif* called DAZ (dif Activation Zone), where oppositely oriented KOPS (FtsK Orienting Polar Sequences) converge and guide FtsK DNA translocation toward the *dif* locus (Pérals et al., 2000). This directional control is achieved by the interaction between the Xer recombinase system and the C-terminal domain of FtsK (Filamentous Temperature-Sensitive cell division protein K), a large division septum-associated DNA translocase, which coordinates chromosome segregation and cell division when chromosome organization has been affected (e.g., chromosome dimer formation, decatenation or delayed replication) (Löwe et al., 2008; Graham et al., 2009; Grainge et al., 2011; Nolivos et al., 2012; Bisicchia et al., 2013; Besprozvannaya and Burton, 2014; Diagne et al., 2014). FtsK was initially documented in 1995 due to observations in *E. coli* TOE44 (AB2497 *ftsK44*) mutant cells and their ability to form long chains of cells due to a single substitution of one amino acid in the N-terminal domain (FtsK_N_), by then, FtsK was thought to participate in septum formation as a peptidoglycan-modifying enzyme. Then, Yu et al. (1998) demonstrated that inactivation of the C-terminal region of FtsK affected normal chromosome segregation due to the formation of long chains of cells and detected abnormal DNA distribution in some *ftsK1::cat minB* double mutants of *E. coli* minicells. Finally, in Steiner et al. (1999) discovered that SSR at dif requires FtsKC and thus, CDR only occurs in its presence. The ∼1329 aa FtsK protein can be divided into three domains; The ∼279 aa N-terminal domain (FtsK_N_) is responsible for attachment of the protein to the membrane by four transmembrane segments and interaction with other proteins of the division septum such as FtsZ (Berezuk et al., 2014). The linker domain FtsK_L_, not commonly conserved in FtsK homologs, is primarily composed of proline-glutamine residues (Dubarry et al., 2010). Its length and composition varies between species, being ∼650 amino acids long in *E. coli* and most of Proteobacteria, ∼200 aa long in *Vibrios* (Val et al., 2008) or ∼125 aa in *Pseudomonas* (Massey et al., 2006). Experiments performed by Bigot et al. (2004) demonstrated that *ftsK_L_* mutations increased filamentation phenotypes even higher than *xer* mutants and that this filamentous formation did not correspond to problems in CDR. On the contrary, it was thought to be due to a deficient positioning of the protein, reducing the possibility of contact between FtsK_C_ and the DAZ region of the chromosome. Subsequently, Dubarry et al. (2010) revealed that different parts of the linker domain interact with other proteins of the divisome such as FtsQ, L, I and Z and these interactions help to stabilize the whole divisome at the site of septation. Interestingly, they also suggested that FtsK_L_ domain may stop or slow down cell division during dimer resolution because of the destabilization of the divisome components when FtsK_C_ has been pulled by the DNA during translocation, this force can separate FtsZ and delay septum constriction. There is also a proportional relation between the glutamine-proline concentration and its length, where the longest linkers are usually richer in these residues (Bigot et al., 2007). Following the linker, the highly conserved ∼500 aa C-terminal domain (FtsK_C_), usually referred as the motor of the FtsK, is comprised of three separated subdomains called α, β and γ. Structural studies of the translocation module FtsK_αβ_ of *Pseudomonas aeruginosa* demonstrated that it assembles as a hexameric ring around double-stranded DNA forming a central channel of 30 Å in diameter, where double stranded DNA (dsDNA) passes through (Massey et al., 2006; Demarre et al., 2013). Later structural studies of the orientation module FtsK_γ_ of *P. aeruginosa* and *E. coli* demonstrated that six γ subdomains are loosely attached by a short linker of 10 aa to the hexameric ring FtsK_αβ_ (Sivanathan et al., 2006; Löwe et al., 2008). The FtsK_αβ_ subdomains are responsible for the ATP hydrolysis-dependent DNA translocation of the protein. The 68 aa FtsK_γ_ subdomain is a helix-wing-helix domain that performs two main functions. The first role of this subdomain is to recognize the 8 bp KOPS sites and then directs FtsK translocation toward the *dif* site located within the *ter* region, at which, if concatenation occurs, two *dif* sites will be brought together to form the synaptic complex XerCD/*dif* (**Figure 2A**). KOPS are over-represented on the leading strand of replication where their concentration gradually increases as *dif* is reached; indeed, more than 90% of KOPS sequences nearby *dif* are located on the leading strand (Nolivos et al., 2012); giving a possible estimate of 34 KOPS motifs located in the DAZ region (Lee et al., 2014) with a frequency of 1 motif every 13Kb (Besprozvannaya and Burton, 2014). The second main function of the FtsK_γ_ subdomain is to activate the XerD catalytic function to generate the first HJ and subsequent dimer resolution (Grainge et al., 2011; Lee et al., 2014; Keller et al., 2016). How FtsK locates and assembles to initiate translocation in the correct KOPS sequence is still arguable, for this reason two models have been proposed; the loading model and the target search model, recently reviewed by Besprozvannaya and Burton (2014). New evidence strongly suggests that FtsK acts in a 350-kb region around *dif* that covers 7% of the genome where monomers of FtsK_C_ assemble exclusively at KOPS motifs as described by the loading model. An initial interaction of a single monomer of FtsK_γ_ will trigger a rapid and stepwise formation of the hexameric ring under high concentration of FtsK (Graham et al., 2010; May et al., 2015). It is likely that FtsKγ assembles quickly and binds to KOPS as a trimer initially, with three FtsKγ modules interacting with consecutive GGG, NA, and GGG bases and then it hexamerizes gradually (Löwe et al., 2008). Once a KOPS motif is detected, allosteric modifications occur leading to hexamerization of FtsK_αβ_, which alters the angular conformation of FtsK_γ_ on the DNA affecting KOPS recognition, and activates FtsK_αβ_ ATP hydrolysis. As a consequence, FtsK is no longer able to recognize subsequent KOPS motifs during translocation, unless FtsK migration is impaired, and KOPS recognition is obligated to restart (Sivanathan et al., 2006; Löwe et al., 2008; Crozat and Grainge, 2010; Lee et al., 2014). FtsK has been demonstrated to be the fastest known DNA translocase, reaching levels of 17.5 ± 3.5 kb/s at 37°C or even faster with a striking stall force and a slight supercoiling induction, 1 positive supercoil per every 150 bp translocated (Saleh et al., 2005; Graham et al., 2009; Lee et al., 2012). It has also demonstrated a striking capacity to displace, evict or bypass different obstacles, especially proteins bound to the DNA such as RNA polymerases (Lee et al., 2014). However, FtsK acts differently upon collision with RecBCD and XerD-XerCD/*dif* complex proteins. When FtsK collides with XerCD/*dif*, in a synapsed form, it activates XerD to create the XerD-HJ transient intermediate (structural rearrangements increase the distance between *dif* sites from about 53 to 67 A°) (Zawadzki et al., 2013; Bebel et al., 2016) followed by a rapid dissociation from the DNA (dissociation takes to 0.5–1 s). Cleverly, May et al. (2015) demonstrated that recombination of the synaptic complex XerCD/*dif* takes 1 s longer than the FtsK dissociation time. Therefore, they suggested that this time span can provide a regulatory control for dimer resolution because concatenated chromosomes will reform XerCD/*dif* synaptic complexes every time that resolution failed. Thus, multiple sets of FtsK hexamers colliding multiple times against XerCD/*dif* synaptic complexes will increase the likelihood of generating recombinant products. This regulatory mechanism ensures monomeric products are formed during translocation of impaired DNA.

## Alternative *dif*/Xer Resolution in Prokaryotes

### Plasmid Resolution: Multicopy Plasmid ColE1 and Accessory Proteins

Plasmid dimerization and eventual multimerization has been termed as the “Dimer catastrophe” due to its deleterious effect in cell populations (Summers et al., 1993; Field and Summers, 2011). Dimer catastrophe represents two major problems in bacteria; (1) unequal plasmid distribution among populations, in particular, multicopy plasmids that are more vulnerable to plasmid loss and (2) metabolic burden caused by the rapid accumulation of dimers into the host (Field and Summers, 2011; Million-Weaver and Camps, 2014). As mentioned previously, dimer resolution was originally elucidated in ColE1, resulting in the first functional characterization of XerC and subsequent identification of XerD by sequence homology to XerC (Blakely et al., 1993). These discoveries constituted a new approach for site-specific recombinases and their role in dimer resolution. Subsequent investigations led to the identification of SSR enzymes involved in dimer resolution in other plasmids of *E. coli*, and other bacteria. Current estimates have identified more than 1300 tyrosine recombinases where many of them are associated with other host proteins to regulate their activity, directionality, or processivity (Meinke et al., 2016). Large plasmids usually carry their own recombinase machineries adjacent to the recombination site. Whereas, small plasmids, like those in the ColE1 family, use the chromosomally encoded dimer resolution system of their host (Sengupta and Austin, 2011; Crozat et al., 2014). For ColE1 resolution, XerC/D proteins act on a specific site called *cer* (**Figure 3B**), a non-codifying region of 280 bp where two additional proteins act with XerCD to catalyze SSR reactions: the arginine repressor (ArgR) (an arginine-dependent DNA binding protein originally called XerA) (Stirling et al., 1988a,b), and aminopeptidase A (PepA) (a bifunctional transcriptional regulatory protein that reacts to environmental signals, which was originally called XerB) (Stirling et al., 1989). SSR in *cer* is catalyzed by XerC within a sequence of 30 bp composed of two 11 bp half sides and a central region of 8 bp. XerC and XerD bind to the left and right halves cooperatively and respectively. Strand exchanges are catalyzed by XerC to form a HJ intermediate that is eventually resolved by an uncharacterized cellular HJ resolvase to generate a recombinant product (Colloms et al., 1996; Cornet et al., 1997). The *cer* site is comprised of a 30 bp core recombination site and two accessory DNA sequences of ∼180 bp in length, in which one or two hexamers of PepA and one hexamer of ArgR control the reaction (Colloms, 2013). The two accessory proteins are necessary for recombination, since in their absence, plasmid dimer resolution cannot be completed. However, at abnormal high concentrations of PepA, recombination *in vitro* can proceed without the help of ArgR (Reijns et al., 2005; Sénéchal et al., 2010). This is also seen at the recombination site *psi* of plasmid pSC101 which requires XerC, XerD and PepA but not ArgR (*psi* dimer resolution requires another accessory protein called ArcA instead of ArgR, and the cleavage reaction is performed by XerC and XerD) (Cornet et al., 1994; Colloms et al., 1996). In *cer*, PepA and ArgR control recombination directionality so that dimers can only be converted into monomers and not the opposite reaction. Dimer resolution directionality caused by these two proteins involves the formation of two directly repeated *cer* sequences positioned in an antiparallel direction; this conformation is favored by negative supercoiling where the *cer* sequences are interwrapped three times around the proteins resulting in the formation of a right-handed synapse structure that brings the XerCD binding sites together. Sites in an inverted repeat position prevent right-handed formation; this ensures only dimer resolution occurs. Thus, XerC and XerD bind to the 30 bp *cer* synapse region and may interact with the N-terminal domains of the PepA hexamers. Whereas the ArgR protein, which is flanked by one or two PepA hexamers, might be involved in bending the DNA, tightening it and activating *cer* SSR by possible interaction with the C-terminal region of the Xer recombinases. Another possible function is to bring the two *cer* sites together and to allow PepA loading to form a nucleoprotein complex that promotes XerCD binding and recombination (Reijns et al., 2005; Sénéchal et al., 2010; Colloms, 2013). Additionally, *cer* also encodes for a 70 nt RNA fragment called Rcd that is only transcribed during dimer formation by the P*_cer_* promoter and regulated in a sequence-specific manner by the FIS protein (Blaby and Summers, 2009). Rcd binds to the enzyme tryptophanase and induces a quiescent state by increasing indole production within the cell. The quiescent state permits the cell to arrest cell division and chromosomal replication but still be active metabolically. This process is thought to be part of a dimer formation checkpoint that allows the XerCD/*cer* system to resolve dimer formation during this pause (Field and Summers, 2011).

### The *Bacillus subtilis* Model and the Effect of Two Translocases

The capacity to perform SSR to resolve chromosome dimers is highly distributed among bacteria and archaea. Thus, homologs of XerC and XerD have been sequenced in a variety of species (Wang et al., 2013; Crozat et al., 2014). In *B. subtilis*, two homologs of XerC and XerD called CodV and RipX perform dimer resolution at a 28 bp *dif* site (*Bsdif*) close to the terminus region (**Figure 3D**). The *Bsdif* region is comprised of two 11 bp half-sites with imperfect dyad symmetry where CodV and RipX bind simultaneously and a 6 bp central region where DNA exchange occurs. Both CodV and RipX share a 37 and 44% identity with the XerC and XerD respectively, and 39% between them (Sciochetti et al., 1999). CodV binds preferentially to the left half-site and preferentially cleaves the top strand whereas RipX is able to bind to both sides with preferential binding to the right-half-site and preferential cleavage of the bottom strand. Cleavage by CodV is more efficient than cleavage by RipX, which suggests that CodV performs the first strand cleavage followed by RipX in *in vitro* experiments (Sciochetti et al., 2001). Sciochetti et al. (1999) also demonstrated that RipX could interact effectively with the *E. coli dif* site, unlike CodV which showed a weaker interaction with this substrate. However, addition of XerC to RipX/*dif_E.coli_* or XerD to CodV/*dif_E. coli_* generated larger complex formation in gel retardation analysis, demonstrating protein-protein interactions between these four proteins, which confirms some conserved features of tyrosine recombinases among bacteria. This is supported by the fact that the right half-site presents highly conserved features with respect to other *dif* sites among some bacteria, whereas the left-half site is less conserved, which could explain why RipX can bind*dif_E. coli_* (Sciochetti et al., 2001). In contrast to *E. coli*, the synaptic complex can be brought together by the action of two DNA translocases: the membrane-associated SpoIIIE protein (Stage III Sporulation Protein E) and the soluble SftA protein (Septum-associated FtsK-like Translocase of DNA). Both translocases harbor AAA+-ATPase and C-terminal domains with 56% of sequence similarity between them. SftA exhibits 50% identity with respect to the *E. coli* FtsK_γ_ domain whereas SpoIIIE exhibits a 50% of similarity to the FtsK_αβ_ subdomain and 42% of similarity to the FtsK_γ_ subdomain of *E. coli* (Barre, 2007). The N-terminal domains of these proteins are more divergent; SpoIIIE and FtsK share 36% of identity to respect to the four transmembrane helix whereas SftA lacks the transmembrane spanning domain (Wu, 2009; Kaimer et al., 2011). N-terminal domain variations coincide with their different location and activation in the genome. FtsK and SpoIIIE share a similar mechanism to anchor to the inner membrane of the dividing cell by their N-terminal regions; this transmembrane interaction is possibly reinforced by interactions with FtsZ or other cell division components such as FtsA or ZapA (Dubarry et al., 2010). During vegetative growth, SpoIIIE shows two predominant states: a static phase, where SpoIIIE is assembled close to future sites of cellular septation, and a mobile phase, where SpoIIIE does not occupy a specific position. Once cellular division begins, the static phase takes place when SpoIIIE is recruited by FtsZ and other division machinery proteins and is escorted to the center of the division septum (Fiche et al., 2013). SpoIIIE remains in the invaginating septum and hexamerizes independently of the cell division stages (Vegetative, division and sporulation stages) and independently of DNA interaction (Cattoni et al., 2014), suggesting that SpoIIIE assembly may not be restricted to the presence of impaired DNA and on the contrary, may be involved in normal DNA segregation as demonstrated by (Fiche et al., 2013). Experiments using high-resolution microscopy revealed that under formation of asymmetric (sporulation) or symmetric (vegetative growth) septa, the SpoIIIE concentration increased 2.5-fold around the constricting septa, even without evident formation of the septa, indicating close interaction with other components of the division machinery, that in turn regulates its activity under specific conditions (Fiche et al., 2013). SftA can be localized either to the cell center or more frequently, to the forming division septum. Although SftA lacks an integral membrane domain, the FtsZ ring recruits the enzyme and attaches it to the division septum during the initiation of cellular division, which explains its localization through the cell cycle (Kaimer et al., 2009, 2011; Kaimer and Graumann, 2011). These patterns of localization suggest that both translocases (SftA and SpoIIIE) are present at the septum at various times of segregation and that they perform DNA migration independently of each other, although SftA is only involved in DNA cytokinesis in contrast to SpoIIIE that may be involved in cytokinesis and cell division processes (Kaimer et al., 2009). DNA translocation is initially carried out by SftA during septation, probably by recognition of the 8-nucleotide SRS motifs (SpoIIIE Recognition Sequences) which are similar to the *E. coli* KOPS sequences. The SRS motifs are mostly located on the leading strand (up to 85%), and direct translocation toward the *Bsdif* site (Besprozvannaya and Burton, 2014). Therefore, the primary function of SftA consists of moving chromosomal DNA until the *ter* regions are positioned at midcell and the origin regions migrate to each pole of the cells. The SftA may also be required for other proteins involved in cytokinesis and FtsZ positioning (Biller and Burkholder, 2009). SpoIIIE, the second translocase in *B. subtilis*, may take over DNA translocation working synergistically but not interchangeably with SftA, it can also function as a DNA segregation checkpoint preventing membrane fusion until chromosome segregation is completed (Kaimer et al., 2011; Fiche et al., 2013). Cattoni et al. (2014) suggested that SpoIIIE binds non-specifically to the DNA in a pre-formed hexameric open ring conformation and then searches for SRS motifs without hydrolysis of ATP. Similarly to FtsK proteins, SRS recognition by the SpoIIIE_y_ domain triggers allosteric modifications that activate the ATPase activity of SpoIIIE_αβ_ and therefore, DNA translocation (Besprozvannaya et al., 2013). Once it encounters SRS motifs, the hexameric ring changes to the closed and active form pumping the chromosome in an oriented manner by recognizing further SRS motifs and translocating it toward *Bsdif* (Cattoni et al., 2014). As mentioned before, SpoIIIE is actively expressed in all growing cells and is essential during sporulation to translocate the remaining DNA from the mother cell into the forespore compartment, and during vegetative growth to guarantee that concatenate formation or disrupted genomes will not affect normal cellular division. Moreover, SpoIIIE is also required for septal membrane fusion after completion of chromosome translocation. During sporulation, asymmetric septation encloses the DNA and traps 25–30% of one chromosome into the forespore. SpoIIIE pumps the remaining 70–75% by an analogous mechanism used by FtsK; the reaction only takes 20 min demonstrating its incredible speed (Demarre et al., 2013; Bose et al., 2016). The mechanism of SpoIIIE DNA translocation through the membrane is still unclear, since recent single molecule-imaging experiments still provide valid information for two main models; the paired DNA conducting channel model (Burton et al., 2010; Yen Shin et al., 2015) and the aqueous channel model (Fiche et al., 2013). This system of both translocases has been also detected in *Staphylococcus aureus* termed FtsK and SpoIIIE because of their amino acid homology to SpoIIIE and FtsK from *B. subtilis* and *E. coli* respectively. However, in contrast to *B. subtilis* system, in *S. aureus* both enzymes seems to present a redundant, although independent role in DNA segregation. Individual deletions of either FtsK or SpoIIIE did not exhibit major changes in chromosome segregation for *S. aureus*, however, when combined together they represented a major threat for *S. aureus* genome stability (Veiga and Pinho, 2016).

Consistent with their different roles, SftA and SpoIIIE do not colocalize during vegetative-replicative stages or sporulation. Thus, SftA in concert with FtsZ and division proteins moves chromosomal DNA away from the closing division septum. Then, upon septum closure, entrapped DNA is translocated through the SpoIIIE pore or channels into the correct compartment (either a forespore or a daughter cell). However, unlike FtsK that activates XerCD recombination reactions, neither SftA nor SpoIIIE directly activate CodV or RipX recombinases. In this case, SftA and SpoIIIE affect the CodV/RipX reaction by proper positioning of the *ter* region, but there is no evidence of direct interaction between these enzymes to date (Biller and Burkholder, 2009; Kaimer et al., 2011).

### Multichromosome Bacteria and IMEX

*Vibrio cholerae*, as well as 10% of sequenced bacteria to date, possess a very distinct property among bacteria; it harbors more than one chromosome (Jha et al., 2012). One ancestral chromosome I (chrI) of 2.96 Mbp and one plasmid-derived chromosome II (chrII) or ‘chromid’ of 1.072 Mbp, encode 2,775 and 1,115 ORFs, respectively. ChrI contains most of the housekeeping genes whereas chrII contains essential genes specialized in adaptation to new environments or pathogenicity (Xu et al., 2003; Harrison et al., 2010; Kirkup et al., 2010; Val et al., 2016). Harboring two or more chromosomes have shown to be highly heritable among these bacteria, which suggests that multiple chromosomes offer a positive selective pressure to maintain them. One possible explanation is that multiple chromosomes might offer an advantageous feature against dimer formation. Val et al. (2008) showed that dimer formation increases exponentially in relation to the size of the replicons, thus, dividing a single replicon into two or more replicons may reduce this topological problem. However, genome size might not be relevant for the presence or absence of Xer/*dif* recombination machinery. Some large chromosomes do not require Xer/*dif* recombination machinery as in some *Legionellales* (genome size ranging from 2 to 5 Mb) whereas some small-sized chromosomes still require Xer/*dif* recombination machinery as demonstrated by some *Rickettsiales* (genome ranging from 0.85 to 1.52 Mb in size) (Carnoy and Roten, 2009).

Homologs of XerC/XerD and FtsK have been characterized on chrI, referred as XerC_V C_ and XerD_V C_ with 53 and 68% of amino acid similarity to *E. coli* XerC and XerD, respectively (Huber and Waldor, 2002; McLeod and Waldor, 2004). Whereas chrII does not encode any Xer recombinase involved in dimer resolution. *dif*-like sequences are present in both chromosomes (*dif1* and *dif2*) located near GC skew shift-points (Val et al., 2008; Kono et al., 2011). Interestingly, both *dif* sites differ from each other in their sequences, *dif2* harbors five different nucleotides compared to *dif1* and most α-proteobacterial *dif* sites, four of them in the central region, resembling *dif*-like plasmid composition (Kono et al., 2011). Dimer resolution in *V. cholerae* requires FtsK_V C_ translocation by recognition of KOPS-like motifs (GGGNAGGG) in a similar way to that found in *E. coli*. Once the *dif* sites are brought together nearby, FtsK_V C_ activates XerD_V C_, which is positioned to cleave the bottom strand, and perform the first strand cleavage. Then XerC_V C_ cleaves the top strand and performs the second strand cleavage; these reactions are carried out on both chromosomes at their respective *dif* sites (**Figure 3E**) (Val et al., 2008). Additional studies demonstrated that *E. coli* FtsK was able to activate 50% of the XerCD_V C_ synaptic complexes at *dif1* whereas only 20% of XerCD_V C_ were activated at *dif2*, suggesting that the *dif2* recombination process requires more accurate interactions between the FtsK proteins and the XerCD complex (Val et al., 2008). An additional feature of multiple chromosomes is their capacity to synchronize replication termination at the same time despite their different sizes (Val et al., 2016). This capacity may confer an additional regulatory control against dimer formation due to the time-lapse between the replicated chromosomes and cellular division. Demarre et al. (2014) showed that *terII* sites (chrII) separate earlier than *terI* and that this early separation keeps *terII* sites at midcell by the macro domain MatP/*matS* organization system. This restriction during concatenation induces several collisions at midcell between *terII* sites, increasing the number of recombinational events and the likelihood of dimer resolution. It also ensures that *ter* sites of bacterial chromosomes remain exclusively in mid-cell to be processed by FtsK.

Although XerC and XerD recombinases normally perform dimer resolution, they are also exploited by other replicons such as plasmids, bacteriophages, and other integrative elements. Indeed, initial studies on plasmid stability in ColE1 and phage integration of bacteriophage λ led to the discovery of XerC and the mechanistic insights of the tyrosine family (Meinke et al., 2016). In *V. cholerae*, the causative agent of the potentially fatal human disease cholera, XerC_V C_ and XerD_V C_ are hijacked by some vibriophages to integrate their genomes into the chromosome. They are usually referred to as IMEX (Integrative Mobile Elements Exploiting Xer), and the best known ones are VGJϕ (Vibrio Guillermo Javier filamentous phage), TLCϕ (Toxic Linked Cryptic), and CTXϕ (Cholera Toxin Phage). CTXϕ is a lysogenic [(+)ssDNA] filamentous bacteriophage that encodes the A-B type enterotoxin CT in *V. cholerae* (Das, 2014). These three vibriophages harbor a particular attachment site (*attP*), a *dif*-like site that serves to classify the three different groups of IMEX, (CTXϕ-type, VGJϕ-type and TLCϕ-type) (Das et al., 2013). Although the components to integrate their genomes are very similar, their mechanisms of integration differ from one to the other and from their host strains. Direct ssDNA integration by CTXϕ-type phages is characterized by the formation of a ∼150 bp folded structure created by the intra-strand base pairing interaction between two palindromic *attP* sites (*attP1* and *attP2*) separated by 90 nt on the ssDNA sequence (**Figure 3F**) (Das, 2014). The two overlap regions *attP1* and *attP2* reassemble the XerC_V C_ side of *dif1* and *dif2* regions but differ from the XerD-side. This lack of homology between XerD_V C_ recognition site and *attP_CTX_*ϕ limits the catalytic reaction to XerC_V C_ that catalyzes the complete reaction. An additional host factor called EndoIII participates in the directionality of the reaction, which blocks further rounds of strand cleavage by XerC_V C_ causing its dissociation and therefore preventing CTXϕ excision (Bischerour et al., 2012). Although XerD_V C_ is not involved in the catalytic reaction, it is still necessary for a successful integration, probably by its role in synaptic complex formation (Val et al., 2005). Once the integration is completed, host DNA replication proteins resolve the formed HJ intermediate and convert it to dsDNA. Prophage CTXϕ cannot be excised from its host since it loses the capacity to fold itself, which in turn prevents further base-pairing interactions between the *attP* sites, which ultimately abolishes the XerC_V C_ catalytic reaction (Das, 2014). Interestingly, CTXϕ integration in El Tor strains is only found in chrI, and it is generally associated with two other vibriophages, TLCϕ and RS1 that enable CTXϕ integration in *V. cholerae* genome by reconstituting a functional *dif* site, and by promoting CTXϕ replication and transmission (Hassan et al., 2010). In the classical biotype strains, CTXϕ usually targets both chromosomes (Faruque and Mekalanos, 2012).

Similarly, to CTXϕ, VGJϕ integration uses the XerC_V C_ catalytic reaction at the *dif1* site, but unlike CTXϕ, it only harbors one *dif*-like attachment site (*attP_V GJ_*ϕ) of 29 bp that allows its integration into the chromosome as a dsDNA. The *attP* central region contains four different nucleotides close to the XerD binding side with respect to the central region of the *dif1* site (**Figure 3G**). The lack of homology at the XerD_V C_ central region side prevents XerD_V C_ participation in the catalytic reaction. Once integrated, prophage VGJϕ acquires two *attP* sites (*attPL* and *attPR*), equally functional for the XerC_V C_ excision reaction, in contrast to CTXϕ, where Xer recombinases can process VGJϕ excision from the host genome (Das et al., 2013). TLCϕ also depends on host encoded Xer recombinases for its integration. Its *attP_TLC_*ϕ site possesses high homology with the XerC_V C_ binding side and central region of *dif1* whereas it is highly divergent from the XerD_VC_ binding site (**Figure 3H**). The prophage form of TLCϕ is almost always linked to CTXϕ integration confirming the regular synergistic interactions found in most IMEX. Paradoxically, despite the lack of homology between the XerD binding sites of *dif1* and *attP_TLC_*ϕ, TLCϕ integration/excision is mediated by XerD_V C_ and then completed by XerC_V C_ resembling dimer resolution in bacteria, but independently of FtsK participation (Midonet et al., 2014).

IMEX are recombination platforms that permit bacteria to evolve and adapt through the acquisition and reordering of relevant genes. They have strengthened bacterial evolution, playing an important role in the rise of multidrug resistance, gene transfer mechanisms and virulence factors among clinically relevant bacteria (Fournes et al., 2016; Midonet and Barre, 2016). Besides the vibriophages just described above, some other relevant IMEX have been found; the gonococcal genomic island (GGI) related to pathogenic *Neisseria* species (Domínguez et al., 2011) and the EludIMEX-1 found in *Enterobacter ludwigii* (Antonelli et al., 2015). GGI is an unusually long IMEX (57 kb long) found in almost 80% of *Neisseria gonorrheae* strains and is involved in the expression of type IV secretion system (T4SS) genes (Christie et al., 2014). GGI carries a degenerate *dif* site called *dif_GGI_* of 28 bp with a XerC-binding site and a central region homologous to the conserved *Neisseria dif* site (*dif_Ng_*) and a divergent XerD-binding site. GGI insertion into the *Neisseria* genome follows a CDR-like process where FtsK activates XerD to perform the first strand cleavage between *dif_Ng_* and *dif_GGI_* followed by isomerisation of the synaptic complex and activation of XerC to perform the second strand cleavage, creating a GGI integrated form with two active Xer binding sites. Interestingly, the GGI synapse has given important clues about how IMEX might remain integrated in the host genome despite the presence of *dif* sites. Fournes et al. (2016) revealed by experiments *in vitro* that a trimeric form of the *E. coli* FtsK protein (t-FtsKαβγ_Ec_) was unable to activate XerCD recombination at one of the two *dif* sites (the *dif_GGI_* site), in fact, the XerCD/*dif_GGI_* complex was unable to stop t-FtsKαβγ_EC_ translocation. As a consequence, the XerCD complex is dissociated from *dif_GGI_* and the excision process is inhibited.

EludIMEX-1 is a 29.1-kb IMEX found in *E. ludwigii* (ECAA-01) that carries the *bla_NMC-A_* gene that encodes for a serine carbapenemase. It was first characterized by Antonelli et al. (2015) when they sequenced the whole genome of a NMC-A-positive isolate of *E. ludwigii*. The results indicated the presence of a new 29-kb region with lower GC content when compared to the bacterial genome, indicating a possible gene transfer acquisition (Wu et al., 2012). Further analysis revealed that this region is flanked by putative XerC/XerD recombination sites with high homology at the XerC-binding site. They also determined that EludIMEX-1 insertion site in the genome was the same for two distinct species of the *E. cloacae* complex suggesting a possible acquisition via a XerC/XerD dependent recombination event at a specific *dif*-like site (Antonelli et al., 2015). Understanding of IMEX control and excision processes will provide us a better idea of how counteract the acquisition of antibiotic resistance genes in pathogenic microorganisms.

### The *difSL*/XerS Model

The *E. coli* pathway of dimer resolution has been found to be highly conserved among bacteria with circular chromosomes. It was initially demonstrated by Recchia and Sherratt (1999) when they analyzed 16 eubacterial and five archaeal genomes for XerCD-CodV/RipX homologs. They showed that most eubacterial genomes possess two putative Xer recombinases whereas Archaea presented a single recombinase in three of the five genomes analyzed (Recchia and Sherratt, 1999). Subsequently, Carnoy and Roten (2009) demonstrated by doing an exhaustive computational analysis of 234 chromosomes from 156 proteobacterial species, that 87.8% of the genomes analyzed presented XerCD-like and *dif*-related sequences. Moreover, Kono et al. (2011) predicted by a recursive hidden Markov model method (including XerCD orthologs) that 578 out of 592 bacterial genomes with a single chromosome and 63 out of 66 genomes with multiple chromosomes presented a *dif*-like sequence. Additionally, they remarked how XerC and XerD are conserved in almost 60–70% of bacterial species, and 85% in proteobacterial species (Debowski et al., 2012). These results among many others led to the general view that the *E. coli* pathway is predominant for dimer resolution. However, dimer resolution machinery or regulation of strand exchange may differ: some processes may require or disregard accessory proteins, others may or may not require activation by translocases, some will be mediated by a XerC-first strand exchange whereas others by XerD-first strand exchange and others may need two recombinases or only one. Among these divergences and unique characteristics for each bacteria to solve dimer formation, the less studied ones are the unconventional single recombinases.

Recchia and Sherratt (1999) first mentioned the presence of single recombinases from the identification of two eubacterial genomes harboring only one Xer homolog. It was later confirmed when Le Bourgeois et al. (2007) demonstrated that some species of *Lactococcus* and *Streptococcus* use an alternative Xer recombination machinery. This new Xer complex is based on a single tyrosine recombinase called XerS (356 aa) that acts on an atypical 31 bp recombination site called *difSL* in the presence of dimers. Unlike *E. coli*, the *xerS* gene is found immediately adjacent to the recombination site *difSL* acting as a single module. The *difSL* site differs from most *dif* sites because of its large central region of 11 bp as opposed to the normally found 6–8 bp in all other *dif* regions (**Figure 3I**) (Leroux et al., 2011). Thus, *difSL* consists of two imperfect inverted repeat sites of different sizes separated by the central region where DNA exchange occurs. The inverted repeat region is one nucleotide longer in *difSL* and contains an extra nucleotide in the middle of the right inverted repeat (TTTTCTTGAAA) versus the left part of the sequence (TTTCCGAAAA). This additional spacing suggests XerS/*difSL* may be biased to favor binding in one-half site over the other. It was later confirmed by Leroux et al. (2011), where they also showed that XerS presented stronger interaction with the left-half site of *difSL* than the right-half site, and a preference for initiating the recombination reaction on the bottom strand of the *difSL* site. These results indicate that, although the *difSL* site is relatively symmetric and XerS is a single tyrosine recombinase, there is a bias for where the proteins initially bind to *difSL* and where they initiate the strand cleavage reaction. Thus, the left-bound monomer could activate the right-bound monomer by bending the DNA or changing the conformation of the second monomer which could explain the preferential cleavage and exchange of the bottom strand. This behavior resembles what XerC displays with weak binding but stronger strand exchange when compared to XerD (Nolivos et al., 2010; Leroux et al., 2011). This intrinsic bias alone cannot control the preference of the directionality of the strand cleavage reaction. The achievement of proper control requires the action of a SpoIIIE-like homolog translocase called FtsK^SL^, a protein of 758 aa in length in *Streptococcus mutans* or 816 aa in *S. agalactiae* with low similarity at the N-terminal region between them. This low similarity does not affect its binding preference to the division septum commonly found in most proteins of the FtsK-HerA superfamily (Le Bourgeois et al., 2007). The C-terminal domain of FtsK^SL^ shows 41% similarity at the amino acid level in relation to FtsK*_E. coli_* with four of the five amino acids similar (QR-GN motif) involved in XerD interaction (Keller et al., 2016). On the other hand, FtsK^SL^ is unable to read *E. coli* KOPS motifs as demonstrated by Nolivos et al. (2012), probably due to the lack of common skewed octamers sequences called Architecture Imparting Sequences (AIMS) in *Firmicutes*, which means that KOPS sequences in *Firmicutes* are not as conserved as in proteobacteria (Hendrickson and Lawrence, 2006). This would also explain the divergence between FtsK_γ_ domains even among *Firmicutes.* Additionally, AIMS found in *Lactococcus lactis* differ in both in length and in sequence from traditional KOPS/SRS motifs, being A-rich heptamer motifs instead of the GC-rich octamer motifs (Nolivos et al., 2012). XerS also lacks critical residues found in XerD to interact with FtsK (residues RQ-QQ). Interestingly, XerS/*difSL* recombination occurs almost in a similar fashion to that of *E. coli*. Both Xer systems require FtsK_N_ localization at the division septum and FtsK_C_ translocation to achieved Xer dimer resolution. Additionally, XerS/*difSL* proved to be functional in *E. coli*, despite the lack of homology in their FtsK proteins. Further analyses on FtsK-Xer interactions are required since the exact mode of action is still speculative (Nolivos et al., 2010).

### The *Helicobacter and Campylobacter* (*difH*/XerH) Model

Studies in *Helicobacter* sp and *Campylobacter* sp led to the discovery of another type of single recombinase called XerH that acts on a recombination site called *difH* in a FtsK-dependent manner. It was shown to be involved in chromosome segregation and possibly dimer resolution in *Helicobacter pylori* (Debowski et al., 2012). XerH (354–362 aa) differs from the traditional XerCD (298 aa) recombinases by its size and protein homology (26% of identity with respect to XerCD). It also shows more similarity to XerS (356 aa) in both the size of the protein and the high degree of homology of their recombination sites (Carnoy and Roten, 2009; Leroux et al., 2013). Another characteristic of XerH and a possible hallmark of single recombinases (XerS and XerH) is that the *difH* sequence is also located near the recombinase-encoding gene, indicating a possible individual genetic module for Xer expression (Le Bourgeois et al., 2007; Carnoy and Roten, 2009). Interestingly, most of the epsilon species of 𝜀-proteobacteria harbor a XerH/*difH* system whereas some other 𝜀-proteobacteria (*Sulfurimonas denitrificans* and *Sulfurovum*) possess a system analogous to the classical XerCD system. Additionally, unlike other tyrosine Xer recombinases, XerH activity appears to be affected by a second Xer recombinase called XerT in *H. pylori* (the TnPZ transposon associated recombinase) since under XerT deletion, *difH* recombination levels increased (Debowski et al., 2012). Recent structural studies showed that the *Helicobacter difH* comprises two highly conserved imperfect inverted binding sites of 11 and 10 bp (AGT**T**ATGAAAA and AAAAGTTTGA) in the left and right sides respectively, separated by a 6–10 bp central region (**Figure 3J**) (Bebel et al., 2016) (Unpublished data suggest a 10 bp central region, Leroux et al., 2013). Two subunits of XerH bind cooperatively to each side with a stronger binding affinity as well as cleavage reaction efficiency in the left half site than the right half site (the outer region in *dif* appears to be determinant in the order of binding and cleavage reactions). The left half site preference is due to stronger interaction between XerH and *difH* left site ΔG = -21.3 kcal/mol compared to the right half site ΔG = -15.4 kcal/mol). The extra nucleotide thymine (T4) in the outer region of the left half site confers a specific hydrogen bond between the left arm and the lysine (K290) of XerH that favors stronger protein–DNA interaction with the other three outermost nucleotides, DNA bending and specific positioning of the nucleophilic tyrosine. Surprisingly, XerH assembly on *difH* does not induce strong DNA bending alone and it seems to require FtsK to generate the required conformational rearrangements to favor XerH DNA exchange (Bebel et al., 2016). Results obtained by Leroux et al. (2013) in *difH* of *C. jenuni* (*difH_camp_*) demonstrated that XerH binding to either the left or right site of *difH_camp_* resulted in similar affinities compared to the full *difH_camp_* site possibly due to the similarity between the outer sequences of both arms. Additionally, XerH binding to *difH_camp_* appears to be less efficient than XerS which suggests that it is less cooperative than XerS/*difSL* system. Additionally, these results contradict XerH binding affinities observed in *H. pylori* by Bebel et al. (2016) and in most tyrosine recombinases involved in chromosome resolution since it did not show any binding preference. On the other hand, unlike binding activity, asymmetrical cleavage reactions by XerH were found with a higher efficiency for bottom-strand substrates than top strand, in agreement with the results of Bebel et al. (2016) XerH recombination was also observed *in vivo* between two *difH_camp_* sites located on the same plasmid; it is also suggested that XerH might be involved in decatenation processes because of the apparent absence of Topo IV proteins in *H. pylori* (Debowski et al., 2012; Leroux et al., 2013). Interestingly, despite *difH_camp_* and *difSL* similarities in the recombination sites, the recombinases do not cross-react (XerH does not bind *difSL* and XerS does not bind *difH_camp_* sites) (Leroux, unpublished).

### The Archaea *dif*/XerA Model

In archaea, chromosome resolution appears to be catalyzed by a single recombinase (XerA) in a FtsK-independent manner that acts on a *dif*-like site located in the replication terminus region (Cortez et al., 2010; Serre et al., 2013). XerA shares a conserved C-terminal domain where the active tyrosine and the conserved catalytic residues (R-K-H-R-[H/W]-Y) reside. XerA proteins are well conserved between the archaeal species analyzed with 85% of sequence similarity. The *xerA* gene location is highly variable in archaea; some species exhibit separated *xerA/dif* sequences whereas some others harbor an individual *xerA/dif* module. Unlike most bacteria, the *dif*-like site is not normally located at 180° from *oriC* and conversely, it is located between 122° and 144° from *oriC* in the analyzed genomes, although the *Methanosphaera stadtmanae* genome showed a *dif*-like site at 180° from *oriC*. *dif*-like sequences consist of the traditional structure; two inverted repeat sequences of 11 bp separated by a central region of 6 bp. XerA catalyzes cleavage reactions without any detectable strand preference (Cortez et al., 2010; Serre et al., 2013).

Although archaea do not require a FtsK homolog to perform chromosome resolution, KOPS-like motifs have been found in Archaea. These KOPS-like motifs consist of four nucleotides (GTTG OR GTTC) called ASPS (Archaea Short Polarized Sequences) that are skewed toward *dif* sites, showing a similar triangle-shaped diagram observed in Bacteria of skew inversion at *dif* sites (Cortez et al., 2010). Serre et al. (2013) have revealed the crystal structure of XerA proteins from *Pyrococcus abyssi*, and Hwa Jo et al. (2016) from *Thermoplasma acidophilum*. Both groups reinforced the idea of *cis*- cleavage reaction by XerA.

## Future Directions

Much information has been gained on site specific recombinases and dimer resolution. This review has highlighted the complexity of *dif*/Xer recombinase systems in prokaryotes and its importance for genome stability and pathogenicity factors. However, many fundamental questions remain unanswered: how do SpoIIIE and SftA from *Bacillus* activate SSR? Moreover, what is the selective advantage of having two chromosome DNA translocases? Additionally, 12% of the studied proteobacterial species do not possess the traditional Xer recombination machinery. Thus, it is still unknown whether these microorganisms lost the Xer recombination system, never acquired it or developed an alternative system to decatenate the chromosomes. It raises the question of how do bacterial cells handle chromosome decatenation without Xer recombinases and *dif*? Is there an alternative recombination system that functions as the *dif*/Xer system? Regarding single recombinases, have they evolved from XerC/XerD recombinases or vice versa, or did they arise from an ancestral recombinase? These and other issues already considered in this review are being gradually addressed by the use of the latest techniques in real-time imaging with super-resolution microscopy. Such as; photo-activated localization microscopy (PALM) and stochastic optical reconstruction microscopy (STORM), plus the use of other techniques as Förster (Fluorecence) resonance energy transfer (FRET), tethered fluorophore motion (TFM), single-molecule Flourescence resonance energy transfer (smFRET), among others. They are providing a powerful blueprint for investigators studying short- and long-range changes in DNA, DNA/protein, and protein/protein interactions. Researchers in SSR systems and protein–protein interactions might be the most direct beneficiaries of these techniques, especially when it is becoming urgent to further understand IMEX insertion and its subsequent influence in antibiotic resistance and bacterial virulence.

## Author Contributions

All the authors contributed equally to the work by providing: Substantial contributions to the conception or design of the work; the acquisition, analysis, and interpretation of data for the work; Drafting the work or revising it critically for important intellectual concepts; Final approval of the version to be published; and Agreement to be accountable for all aspects of the work in ensuring that questions related to the accuracy or integrity of any part of the work are appropriately investigated and resolved.

## Conflict of Interest Statement

The authors declare that the research was conducted in the absence of any commercial or financial relationships that could be construed as a potential conflict of interest.
